# Optimizing Antihypertensive Management for Hypertensive Patients With Secondary Complications: A Systematic Review and Meta-Analysis in Primary Care Settings

**DOI:** 10.7759/cureus.45834

**Published:** 2023-09-23

**Authors:** Sulaiman Althuwaikh, Ibrahim Albassam, Abdulrahaman Alrashed, Fahad Alhaji, Ahmed Al-Adawi, Mohammed A Sindi, Ahmad Alhibshi, Ahmad Al Dehaini, Layal Alqaysi

**Affiliations:** 1 Internal Medicine, Al-Amiri Hospital, Kuwait City, KWT; 2 Internal Medicine, Lincoln County Hospital, Lincoln, GBR; 3 General Practice, University of Glasgow, Glasgow, GBR; 4 Internal Medicine, University of Glasgow, Glasgow, GBR; 5 General Dentistry, King Abdulaziz University, Jeddah, SAU; 6 Internal Medicine, King Abdulaziz University Hospital, Jeddah, SAU; 7 Internal Medicine, University of Jordan, Amman, JOR

**Keywords:** hypertension, secondary prevention, primary care, blood pressure control, systematic review and meta-analysis

## Abstract

Despite significant pharmacological advancements, hypertension management remains challenging, with varying quality of primary care. Digital tools and other non-pharmacological interventions hold promise in addressing this challenge. Consequently, a thorough examination of these interventions is recommended. This meta-analysis focuses on clinician-oriented strategies aimed at improving hypertension management, to assess the most effective approaches for improving antihypertensive prescribing and blood pressure control for secondary prevention. This was done through a systematic review of randomized controlled trials published in PubMed and Embase since the beginning of 2010 that aimed to enhance antihypertensive medication prescription in primary care settings for hypertensive patients with secondary complications while reporting changes in blood pressure or target achievement. We screened 6305 records. Four studies met the inclusion criteria, with reported interventions including physician education and the implementation of electronic decision support systems. All studies showed that the control group had a statistically significant lower systolic blood pressure, but the effect on diastolic blood pressure was not statistically significant. The overall mean difference was 2.12 mmHg (95% CI = 0.98; 3.26, P-value = 0.0003) for systolic blood pressure in favor of the control group and 1.22 mmHg (95% CI = -0.48; 3.26, P-value = 0.16) for diastolic blood pressure, which was not statistically significant. Despite considerable efforts to control hypertension, it remains a significant obstacle to optimal cardiovascular risk reduction. This review is also limited by a scarcity of studies.

## Introduction and background

Hypertension, often referred to as the "silent killer," continues to loom ominously in the realm of contemporary medical challenges. the high prevalence of hypertension, coupled with its associated comorbidities and mortalities, is a major public health challenge that modern medical science endeavors to combat. In this persistent pursuit, the early identification and proactive intervention for hypertensive individuals, as well as those predisposed to its development, remain one of the cornerstones of public health endeavors [1]. Over the past eight decades, pharmacological interventions have undeniably made significant strides in the management of hypertension [2]; nevertheless, despite sincere and varied efforts, including clinical advancements and various disease-centered campaigns, achieving optimal blood pressure control remains challenging, as only 25% of patients with hypertension have their blood pressure under control, and only 40% of patients with hypertension have their blood pressure below the recommended level after three years of treatment [3].

Taking the forefront against this debilitating disease are primary care centers and the dedicated cohort of primary care physicians. As evidenced by studies conducted in developed countries, a staggering 90% of the adult population engages with their primary care physician at least once annually, underscoring the pivotal role these healthcare professionals play in both hypertension prevention and treatment programs [4]. However, despite their crucial role, ensuring that the recommended standards of care are met is not guaranteed. Various factors linked to primary care centers and the healthcare providers working there can lead to care quality that falls short of expected standards [5].

Non-pharmacological approaches offer a promising path for hypertension management. These methods focus on improving care by primary care physicians and on enhancing patient education and lifestyle choices [5,6]. Computer programs are one tool that can play a key role in detection, diagnosis, and medication. Digital tools are not new, as seen in Finland and Belgium. Meanwhile, discussions on effective education for primary healthcare practitioners are growing, shedding light on modern hypertension strategies [7,8].

Hypertension is associated with several serious complications. These include renal disease, which can lead to kidney damage or failure; all-cause mortality, indicating an increased risk of death from various causes; stroke, a sudden disruption of blood flow to the brain; combined cerebrovascular disease (CVD), encompassing conditions like angina and myocardial infarctions; combined coronary heart disease (CHD), indicating a broad range of heart and blood vessel-related issues [2,6]. Managing hypertension is crucial in reducing the risk of these severe health outcomes.

A systematic review by Fahey et al. has meticulously investigated the potency of educational and organizational interventions in hypertension control. Their findings resonate in highlighting that systematically designed platforms for continuous assessment of antihypertensive regimens have resulted in noteworthy reductions in blood pressure levels [5]. Conversely, disparate intervention approaches have led to varying degrees of success, collectively underscoring the capacity to mitigate high blood pressure. Another pivotal systematic review by Glynn et al. supported this discourse, affirming the efficacious role of both self-monitoring mechanisms and orchestrated systems in counteracting hypertension [6]. Yet, Given the complex body of research, it's crucial to keep analyzing it consistently. To that end, we aimed in this systematic review and meta-analysis to assess interventions that focus on doctors or practices to improve how primary care handles antihypertensive prescriptions. Our goal is to enhance these approaches and refine hypertension management.

## Review

Materials and methods

A systematic review and meta-analysis were conducted as per the Preferred Items for Systematic Reviews and Meta-Analyses (PRISMA) guidelines [9].

Eligibility Criteria

We included randomized controlled trials published from 2010 onwards that compared new interventions to routine care for optimizing blood pressure (BP) control in primary care settings for hypertensive patients with secondary complications. We excluded studies where interventions solely focused on patients’ education or involved community health workers. Additionally, we excluded studies that focused on telemedicine, collaboration with specialists, pay-for-performance, or home blood pressure monitoring. Studies that involved pregnant patients, active controls, or did not report blood pressure change or control were also excluded. Healthcare delivery and feasibility studies were not eligible for this study.

Primary Outcome

The primary outcome is to identify interventions that can optimize blood pressure control for hypertensive patients with secondary complications in primary care settings, compared to usual care.

Literature Search Strategy

The databases used in this systematic review are MEDLINE and Embase through the OVID platform. Citation tracking from the 2010 Cochrane review that evaluated the interventions to improve antihypertensive medication prescribing was conducted [10]. Thesaurus headings, search operators, and limits in each of the above-mentioned databases were adapted accordingly. Language restrictions were applied in our search strategies to English Only. The search using the current search strategy was conducted on the 23rd of May, 2023. Appendix 1 details the search strategy used in each database.

Selection of Studies

The title and abstract of articles identified from the literature searches and citation tracking were assessed independently by four authors. The full texts of relevant articles reports were retrieved and those articles that met the eligibility criteria were selected. A fifth author adjudicated where authors who screened the articles disagreed on a report’s eligibility.

Data Extraction and Management

The data extraction form was based on the Cochrane data collection form for intervention reviews. The data extraction spreadsheet followed Cochrane’s data collection form for intervention reviews. The data extraction form was tested against randomly selected articles and adjusted accordingly. The two authors independently assessed the risk of bias in randomly selected articles using Cochrane's risk of bias tool for randomized controlled trials. A third author resolved any disagreements. The data extraction form included study-related data, baseline characteristics, the outcome of interest data as well as our assessment of their risk of bias. The study-related data included first author, year of publication, the country where the study was conducted, journal in which the study was published, study design, study size, the clinical condition of the study participants, type of intervention and comparison. Baseline characteristics included participants' age, gender, ethnicity, systolic blood pressure (SBP) and diastolic blood pressure (DBP), and the percentage of controlled blood pressure at the beginning of the study. The outcome of interest data included SBP and DBP post-intervention, mean change in their respective mean as well as the percentage of controlled blood pressure at the end of the study.

Data Synthesis

Review Manager 5.4.1 software and Robvis, a web-based application for visualizing risk of bias assessment, were used for data synthesis. We used a random effects model to analyze the data. We reported the results as mean SBP and DBP post-intervention using a Forest plot for the four meta-analyzable studies.

Assessment of Heterogeneity

Heterogeneity was assessed using the Cochrane Q test (χ^2^). The I^2^ statistic was calculated to quantify the inconsistency between the studies. The I^2^ statistic is a measure of the proportion of the total variability in the effect estimates that is due to heterogeneity rather than sampling error. Cochrane’s guide was used for (I^2^) interpretations. The guide suggests that heterogeneity values between 0% and 40% might not be of importance and considers (I^2^) values between 75% and 100% as a considerable heterogeneity range. However, the guide states that (I^2^) is reliant on the magnitude and direction of effects as well as the strength of heterogeneity evidence and hence (I^2^) should be interpreted while considering these factors [11].

Results

Literature Search Result

**Figure 1:**
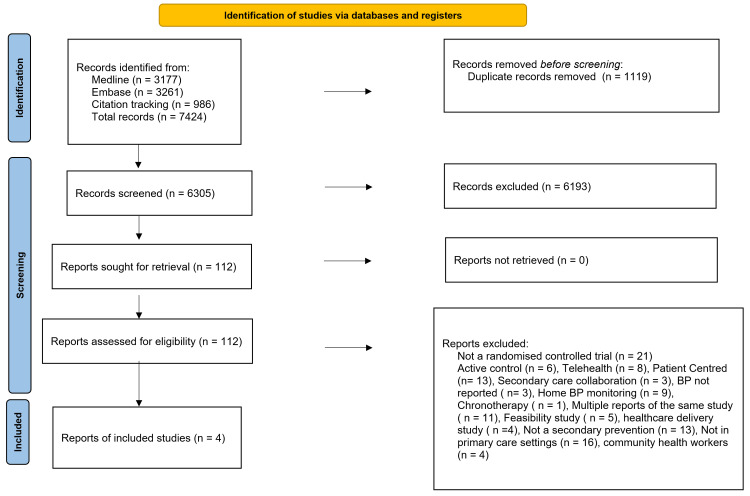
Preferred Reporting Items for Systematic Reviews and Meta-Analyses (PRISMA) diagram. Illustration of articles identification and screening steps.

Our search identified 7424 articles. After deduplication, 6305 articles remained for screening. After the initial screening process, we retrieved 112 articles to further assess eligibility. Only four of the 112 articles were included and the rest were excluded as illustrated in Figure 1.

Description of Studies

Heselmans et al. (2020):This is a cluster randomized controlled trial conducted in Belgian primary care practices between 2017 and 2018. The study investigated the effectiveness of an evidence-based medicine electronic decision support system in controlling hemoglobin A1C (HbA1C), low-density lipoprotein (LDL) cholesterol, and blood pressure compared to usual care. At the end of the study, the data of 3815 of the participants was analyzed [12].

Jafar et al. (2015):This is a multi-center randomized controlled trial conducted in Pakistan. The study investigated the effect of GP education on blood pressure control in patients with hypertension, using the British Hypertension Society guidelines for the Indo-Asian population and the Joint National Committee's seventh report. The intervention continued for two years, and the sustainability of the effect was assessed five years after the study. At the end of the seven years, the data of 632 patients was analyzed. The other two intervention groups were excluded from the review because they met the exclusion criteria [13].

Johnson et al. (2011):This is a randomized controlled trial conducted in the USA and compared physician education, patient education, and combined physician and patient education to usual care in African Americans with hypertension. The patients were followed up for six months. The data of 235 patients from the physician education and usual care arms was analyzed. The data from the other two arms was excluded because they met the exclusion criteria [14].

Pouchain et al. (2013):This is a multi-center randomized controlled trial conducted in France. The trial recruited patients with hypertension and followed them up for 24 months. The study compared the effect of usual care and GP education on the ability of patients to reach the therapeutic targets recommended by the French guidelines. Patients who did not reach the target were followed up for an additional six months. The general practitioners were also provided with a six-page leaflet summary of therapeutic targets and patient encouragement strategies which is to be kept by the general practitioners on their office desks. The outcome of 1832 patients were reported at the end of the study [15].

Changes in BP Control

**Figure 2:**
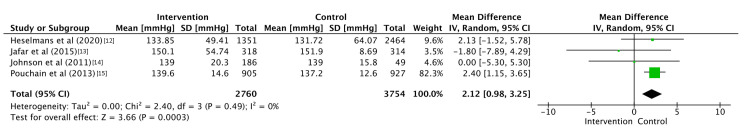
Meta-analysis of systolic blood pressure control

**Figure 3:**
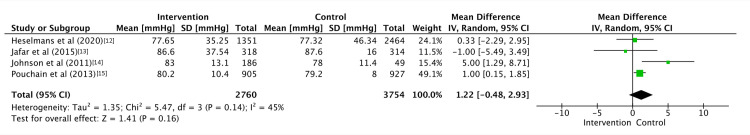
Meta-analysis of diastolic blood pressure control

We assessed how different interventions can help optimize both systolic and diastolic blood pressure control. Figure 2 illustrates the impact of interventions on SBP control, involving 6491 participants. The four studies included reported SBP and the mean difference in SBP is 2.12mmHg (95% CI= 0.98; 3.26, P-value = 0.0003) in favor of the control. The included studies had a low heterogeneity level (I^2^=0%, P-value= 0.49) in terms of SBP. Figure 3 illustrates the impact of interventions on DBP control, involving 6491 participants. The four studies included reported DBP and the mean difference in DBP is 1.22mmHg (95% CI= -0.48; 3.26, P-value = 0.16) which is not statistically significant. The included studies had a moderate heterogeneity level (I^2^=45%, P-value= 0.14) in terms of DBP.

**Figure 4:**
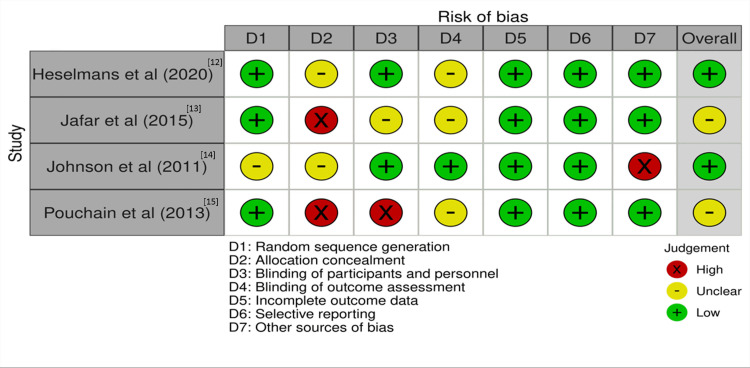
Risk of bias assessment of the included studies

Following the use of the Cochrane risk assessment tool for randomized controlled trials, half of the studies had a low risk of bias overall. The other half had an unclear risk of bias. Furthermore, the studies included had a common feature of either having unclear or high risk of bias in terms of allocation concealment. However, they all had low risk of bias in terms of incomplete data reporting and selective reporting, as shown in Figure 4.

Discussion

The four studies included in this review showed an unexpected result of lowering SBP in the control groups. This might be explained by the differences in recommendations specifying the choice of anti-hypertensive medications, which primarily target improving comorbidity-specific parameters rather than blood pressure numerical targets [16]. For example, in chronic kidney disease and heart failure, initiation with a combination therapy, including a renin-angiotensin-system (RAS) blocker and a diuretic or calcium channel blocker (CCB), regardless of ethnicity and particularly in cases of resistant hypertension, is of utmost importance in reaching blood pressure targets. Given their tendency to accumulate fluids, however, the four included studies stress the importance of angiotensin-converting-enzyme/angiotensin receptor blockers (ACE-I/ARB) in the general population and CCB in the black ethnicity [12-16]. Another possible explanation is the different educational materials used for healthcare workers and patients, which are varied among the four studies included in which the Joint National Committee on Prevention, Detection, Evaluation, and Treatment of High Blood Pressure (JNC 7) guidelines were the backbone of the educational courses given to general practitioners in both Jafar et al. (2015) and Johnson et al. (2011), the French Guidelines for Pouchain et al. (2013), and the EBMeDS system for Heselmans et al. (2020) [12-15]. Moreover, the educational material might favor the lifestyle and be well catered towards the primary hypertension population and in contrast might be sub-optimal in participants with chronic kidney disease and/or heart failure. This can be seen, for example, in the non-pharmacological strategies, where the goal is set to embrace a Dietary Approaches to Stop Hypertension (DASH) diet, reduce salt and alcohol intake, lose weight, and engage in physical activity which are all of proven benefit; however, in heart failure and chronic kidney disease, total daily fluid intake, not accounted for in the JNC 7 guidelines, also plays a major role in blood pressure control [17].

There is also the proposition of the need to implement both interventions to control blood pressure and the accompanying secondary complications, such as chronic kidney disease and heart failure, in order to achieve numerical blood pressure targets. This is supported by the fact that diabetes and hypertension pathophysiology and metabolic pathways intertwine at insulin resistance where the glucose deposition in small blood vessel walls at the collagenous layers can reduce the elasticity of the blood vessels and smooth muscle cell proliferation and thus increase blood pressure [18]. Furthermore, the co-existence of hypertension and dyslipidemia synergizes to increase cardiovascular risk, thus leading to adverse events that further exacerbate blood pressure control [19].

The evidence presented here suggests that we need to research interventions that target hypertension and its secondary complications in order to reach lower blood pressure targets and thus lower all-cause mortality and major adverse cardiovascular events (MACE). This study has a limitation in that it only looked at blood pressure control in patients who already had secondary complications, rather than preventing them from developing in the first place.

## Conclusions

In our systematic review and meta-analysis, the interventions aimed at optimizing blood pressure control for secondary prevention in primary care settings were less effective than traditional care. We found that traditional care was better at lowering systolic blood pressure than the interventions, while the difference in diastolic blood pressure was not statistically significant. Our study was limited by the small number of studies that targeted secondary prevention and hence the inability to generalize whether a particular physician-focused intervention is superior to routine care or not. More research is needed to determine the effectiveness of interventions for secondary prevention of hypertension.

## Appendices

**Table 1:** Databases search strategy

Medline search strategy	
Number	Search terms
1	exp Essential Hypertension/ or exp Hypertension/
2	exp Antihypertensive Agents/
3	(anti-hypertensive* or anti hypertensive* or antihypertensive).mp. [mp=title, abstract, original title, name of substance word, subject heading word, floating sub-heading word, keyword heading word, organism supplementary concept word, protocol supplementary concept word, rare disease supplementary concept word, unique identifier, synonyms]
4	hypertension.mp. [mp=title, abstract, original title, name of substance word, subject heading word, floating sub-heading word, keyword heading word, organism supplementary concept word, protocol supplementary concept word, rare disease supplementary concept word, unique identifier, synonyms]
5	((blood pressure or BP) adj2 (lower* or reduc* or elevat* or increas* or rais* or rise or rising or high)).mp. [mp=title, abstract, original title, name of substance word, subject heading word, floating sub-heading word, keyword heading word, organism supplementary concept word, protocol supplementary concept word, rare disease supplementary concept word, unique identifier, synonyms]
6	1 or 2 or 3 or 4 or 5
7	exp Randomized Controlled Trial/
8	randomi?ed controlled trial.pt.
9	controlled clinical trial.pt.
10	randomi?ed.ab.
11	placebo.ab.
12	drug therapy.fs.
13	randomly.ab.
14	trial.ab.
15	groups.ab.
16	(random$ adj3 (allocat$ or assign$ or basis or order$)).ab,ti.
17	((singl$ or doubl$ or trebl$ or tripl$) adj3 (blind$ or mask$)).ab,ti.
18	(cross over or crossover).ab,ti.
19	7 or 8 or 9 or 10 or 11 or 12 or 13 or 14 or 15 or 16 or 17 or 18
20	6 and 19
21	((((healthcare or health care or health-care) adj professional*) or clinician* or physician* or doctor* or nurs* or pharmacist*) adj3 (intervention* or automated or educat* or train* or teach* or quality or practice or prompt* or feedback or behaviour* or behavior* or decision support* or reminder* or information system*)).mp. [mp=title, abstract, original title, name of substance word, subject heading word, floating sub-heading word, keyword heading word, organism supplementary concept word, protocol supplementary concept word, rare disease supplementary concept word, unique identifier, synonyms]
22	exp Education, Medical/
23	21 or 22
24	exp Primary Health Care/
25	exp Family Practice/
26	exp General Practice/
27	exp Ambulatory Care/
28	exp Patient Care Management/
29	exp Management Information Systems/
30	exp Decision Support Systems, Clinical/
31	exp Reminder Systems/
32	24 or 25 or 26 or 27
33	28 or 29 or 30 or 31
34	32 and 33
35	((primary health care or primary healthcare or primary care or ambulatory care or general practice or general practitioner* or GP*) adj3 (intervention* or automated or educat* or train* or teach* or quality or practice or prompt* or feedback or behaviour* or behavior* or decision support* or reminder* or information system*)).mp. [mp=title, abstract, original title, name of substance word, subject heading word, floating sub-heading word, keyword heading word, organism supplementary concept word, protocol supplementary concept word, rare disease supplementary concept word, unique identifier, synonyms]
36	34 or 35
37	23 or 36
38	20 and 37
39	limit 38 to english
40	limit 39 to yr="2010-current"
41	limit 40 to "humans only (removes records about animals)
Embase search strategy	
Number	Search terms
1	exp hypertension/ or exp essential hypertension/
2	exp antihypertensive agent/
3	(anti-hypertensive* or anti hypertensive* or antihypertensive).mp. [mp=title, abstract, heading word, drug trade name, original title, device manufacturer, drug manufacturer, device trade name, keyword heading word, floating subheading word, candidate term word]
4	hypertension.mp. [mp=title, abstract, heading word, drug trade name, original title, device manufacturer, drug manufacturer, device trade name, keyword heading word, floating subheading word, candidate term word]
5	((blood pressure or BP) adj2 (lower* or reduc* or elevat* or increas* or rais* or rise or rising or high)).mp. [mp=title, abstract, heading word, drug trade name, original title, device manufacturer, drug manufacturer, device trade name, keyword heading word, floating subheading word, candidate term word]
6	1 or 2 or 3 or 4 or 5
7	exp randomized controlled trial/
8	randomi?ed.ab.
9	placebo.ab.
10	randomly.ab.
11	trial.ab.
12	groups.ab.
13	(random$ adj3 (allocat$ or assign$ or basis or order$)).ab,ti.
14	((singl$ or doubl$ or trebl$ or tripl$) adj3 (blind$ or mask$)).ab,ti.
15	(cross over or crossover).ab,ti.
16	7 or 8 or 9 or 10 or 11 or 12 or 13 or 14 or 15
17	6 and 16
18	((((healthcare or health care or health-care) adj professional*) or clinician* or physician* or doctor* or nurs* or pharmacist*) adj3 (intervention* or automated or educat* or train* or teach* or quality or practice or prompt* or feedback or behaviour* or behavior* or decision support* or reminder* or information system*)).mp. [mp=title, abstract, heading word, drug trade name, original title, device manufacturer, drug manufacturer, device trade name, keyword heading word, floating subheading word, candidate term word]
19	exp medical education/
20	18 or 19
21	exp primary health care/
22	exp general practice/
23	exp ambulatory care/
24	21 or 22 or 23
25	exp information system/
26	exp decision support system/
27	exp reminder system/
28	25 or 26 or 27
29	24 and 28
30	((primary health care or primary healthcare or primary care or ambulatory care or general practice or general practitioner* or GP*) adj3 (intervention* or automated or educat* or train* or teach* or quality or practice or prompt* or feedback or behaviour* or behavior* or decision support* or reminder* or information system*)).mp. [mp=title, abstract, heading word, drug trade name, original title, device manufacturer, drug manufacturer, device trade name, keyword heading word, floating subheading word, candidate term word]
31	29 or 30
32	20 or 31
33	17 and 32
34	limit 33 to english
35	limit 34 to yr="2010-current"
36	limit 35 to "humans only (removes records about animals)
